# Uncovering the Role of Selenite and Selenium Nanoparticles (SeNPs) in Adolescent Rat Adipose Tissue beyond Oxidative Balance: Transcriptomic Analysis

**DOI:** 10.3390/antiox13060750

**Published:** 2024-06-20

**Authors:** Fátima Nogales, Eloísa Pajuelo, Inés Romero-Herrera, Olimpia Carreras, Francisco Merchán, José A. Carrasco López, María Luisa Ojeda

**Affiliations:** 1 Departamento de Fisiología, Facultad de Farmacia, Universidad de Sevilla, 41012 Sevilla, Spain; fnogales@us.es (F.N.); iromero3@us.es (I.R.-H.); olimpia@us.es (O.C.); 2 Departamento de Microbiología y Parasitología, Facultad de Farmacia, Universidad de Sevilla, 41012 Sevilla, Spain; epajuelo@us.es (E.P.); fmerchan@us.es (F.M.)

**Keywords:** adolescent rats, adipose tissue, selenium, nanoparticles, transcriptomics, oxidative stress, catabolism, immune system

## Abstract

Studies on adolescent rats, when body composition is changing deeply, reveal that the administration of sodium selenite and selenium nanoparticles (SeNPs), at the same dose, have opposite effects on adipogenesis in white adipose tissue (WAT). To investigate the mechanisms involved in these contrasting effects by means of transcriptomic analysis, three groups of male adolescent rats (*n* = 18) were used: control (C), selenite supplemented (S), and SeNPs supplemented (NS). Both treated groups received a twofold increase in Se dose compared to the control group through water intake for three weeks. Following treatment, WAT was removed and frozen at −80 °C until subsequent use for RNA extraction, endogenous antioxidant enzymatic activities determination, and quantification of H_2_O_2_ and malondialdehyde. NS rats displayed a larger number of differentially expressed genes and cellular processes impacted than S rats. Remarkably, these changes involved upregulation of gene expression associated with the immune system, catabolism, mitochondrial function, and oxidative balance. NS rats presented an increase in antioxidant enzymes activity, alongside an accumulation of H_2_O_2_ and malondialdehyde levels. The expression level of 81 genes related to oxidative stress was significantly affected in NS rats. Analyzing the KEGG pathway enrichment revealed that NS rats exhibited increased activity in key catabolic pathways and decreased activity in crucial growth signaling processes. These changes contribute to the mass decrease in WAT found in NS rats. These results suggest a possible application of SeNPs in WAT reduction and induction of the immune response during adolescence.

## 1. Introduction

Selenium (Se) is a well-known antioxidant trace element with important anti-inflammatory properties mediated by different selenoproteins such as glutathione peroxidases (GPx) [1]. Moreover, it has been recently described as an important mineral related to cell growth and endocrine function [2,3]. This is in part because correct oxidative balance is deeply related to the endocrine signaling process and cell proliferation and differentiation [4]. Currently, it is established that the 25 known selenoproteins intricately regulate the functioning of the endocrine system and intracellular signaling [5]. Antioxidant activity of selenoproteins GPx1, GPx3, GPx4, and thioredoxin reductases (TXNRDs), as well as selenoproteins SELS, SELW, located within the endoplasmic reticulum, and the hepatokine SELP [6], is implicated in this. However, the involvement of Se is not well elucidated.

In this context, this research group has recently found that Se levels are deeply related to white adipose tissue (WAT) metabolism during rats’ adolescence [7]. Nevertheless, its effects clearly differ depending on the administration form. Supplementation with Se at doses below the daily upper limit in the form of selenite contributed to the increase in WAT adipogenesis by stimulating the insulin signaling pathway and by modulating the adipokine secretion profile, especially decreasing lipocalin-2 (LCN2); however, no obesity nor insulin resistance (IR) process appeared. By contrast, the administration of the same doses in the form of Se nanoparticles (SeNPs), which have a high surface area, solubility, and thermal resistance, along with slow excretion rate, sustained release and well-proven anti-inflammatory activity [8,9], and prevented fat deposits in WAT. SeNPs supplementation decreased the insulin signaling pathway and increased the forkhead box O3a (FOXO3a) in WAT, affecting autophagy and lowering inflammation. Interestingly, both forms of Se administration increased the activity of the antioxidant enzyme GPx1 in the same way, indicating that their contrasting effects on WAT mass are GPx1-independent. Therefore, there must be additional factors that contribute to these deep changes in lipid homeostasis. Given the potential for these findings to inform dietary interventions aimed at preventing obesity and/or anorexia during adolescence, a critical period characterized by intense growth and endocrine changes [2,3], further research is needed in this context. Notably, the prevalence of both obesity and IR is on the rise in adolescents [10], with a concerning co-occurrence with anorexia nervosa [11].

The microbiota–liver–bile salts secretion axis is related to lipid homeostasis and WAT development, since it is a significant agent in the maintenance of energy homeostasis and host immunity, modulating WAT mass, obesity, and IR [12,13,14,15]. The communication between the gut and the liver and the adipose tissue works bi-directionally [16]. Therefore, the liver and the WAT also communicate with the intestine and the gut–microbiota axis by secreting different organokines, and in the case of the liver, also, by secreting bile salts [16,17,18,19]. Since Se plays an important role in the maintenance of gastrointestinal tract health by enhancing its antioxidant function, modulating autophagy and apoptosis pathways [20], modulating gut microbiota [21], and affecting primary bile acids biosynthesis in the liver [22], its effects modulating this axis were also explored. Using the same supplementation protocol in adolescent rats [7,23], selenite improved the bile salts profile to stimulate intestinal fat absorption. It also enhanced the ratio of *Firmicutes/Bacteroidetes* directly related to WAT mass, and the incretin glucagon-like peptide-1 (GLP-1) secretion. In contrast, SeNPs supplementation primarily impacted the gut microbiota, inducing a shift towards a Gram-negative dominant profile. This was characterized by a marked increase in the relative abundance of *Akkermansia* and *Muribaculaceae*, accompanied by a decrease in the *Firmicutes*/*Bacteroidetes* ratio. This bacterial profile is directly related to lower adipose tissue mass [24].

It is clear that selenite and SeNPs supplementation effects on WAT homeostasis during adolescence are multifactorial and powerful; therefore, a deeper understanding of the underlying mechanisms is required. For this reason, the aim of this study is to analyze the transcriptome changes in adolescent rats WAT triggered after selenite and SeNPs supplementation, in order to have an integral view of its biological effects on this tissue.

## 2. Materials and Methods

### 2.1. Animals

In this experiment, we used 18 adolescent male Wistar rats from the Centre of Animal Experimentation of University of Seville, located at “Centro de Investigación Tecnología e Innovación de la Universidad de Sevilla” (CITIUS III). These rats were received at 21 days of age, housed in groups of two rats/cage for one week, in a 12:12 light/dark cycle and automatically controlled temperature (22–23 °C), to acclimate them to housing. They were humanely handled, and efforts were made to minimize the number of animals used adhering to the principle of 3R: replacement, reduction, and refinement, as set out in ethical guidelines, whenever possible. The procedures and experimental protocols concerning the protection of experimental animals were in accordance with the guidelines of the European Union Council (Directive 2010/63/UE) and the Spanish Royal Decree (BOE 34/11370, 2013). The research protocol was approved by both the Ethics Committee of the University of Seville (CEEA-US2019-4) and the Junta de Andalucía (05-04-2019-065).

The experimental protocol began on the 28th postnatal day (PND) when the rats reached adolescent stage [25]. At that moment, the rats having similar body weight (49.8 ± 3.3 g) were randomly divided in control (C), sodium selenite supplemented (S) and SeNP supplemented (NS) groups. All groups had access to the standard pellet diet (LASQCdiet^®^ Rod14-R; Märkische, Germany) containing 0.2 ppm of Se in the form of Na_2_SeO_3_, ad libitum. However, S and NS groups received an additional Se supplementation (0.14 ppm) as anhydrous sodium selenite (Panreac, Barcelona, Spain) or SeNPs in the drinking water. The total selenium intake was determined by multiplying the known selenium concentrations in the diet (0.2 ppm) and drinking water (0.14 ppm for both S and NS groups) by the daily amounts of food and water consumed. This treatment was carried out for 3 weeks until PND 47, when the adolescent stage for Wistar rats ends [25]. With the supplementation, the rats consumed on average 6 µg/day of Se, which was equivalent to 500 μg/kg of dietary Se, consistent with the highest GPx activity in rat plasma and liver [26].

### 2.2. SeNPs Synthesis

SeNPs were synthesized at the Department of Organic and Medicinal Chemistry, Faculty of Pharmacy, University of Seville, Spain, using Se tetrachloride (SeCl_4_), ascorbic acid (C_6_H_8_O_6_), and poly(sodium 4-styrenesulfonate) (PSSS) (Sigma-Aldrich, Madrid, Spain). SeNPs were freshly prepared following a previously described procedure [7,27], using optimal amounts of ascorbic acid as a chemical reductant. The precipitation of SeNPs was performed to achieve nanoparticles of the smallest size (less than 50 nm).

### 2.3. Sample Harvesting and Tissue Homogenization

At end of treatment, after fasting all night (12 h) using individual metabolic cages, adolescent rats were anesthetized with an i.p. injection of 28% w/v urethane (0.5 mL/100 g of body weight) and sacrificed by cardiac puncture. The WAT was removed, weighed, frozen in liquid nitrogen, and stored at −80 °C prior to biochemical determinations. The somatic index of this tissue (WATSI) was calculated by dividing the organ weight by the total animal weight.

WAT from each rat (*n* = 6 rats/group) was individually homogenized in 50 mM phosphate buffer, pH 7.0 (K_2_HPO_4_ 50 mM, KH_2_PO_4_ 50 mM, EDTA 0.01 mM (Sigma-Aldrich, Madrid, Spain), protease inhibitor 1:10 (Complete protease inhibitor cocktail tablets, ROCHE, Madrid, Spain)) following routine protocols in our laboratory [7]. The protein content in these homogenates was analyzed by the method of Lowry et al. [28].

### 2.4. Oxidative Balance in WAT

#### 2.4.1. Activity of Antioxidant Enzymes

The activity of superoxide dismutase (SOD), catalase (CAT), and GPx was assayed in WAT homogenates following the methods previously described [29]. GPx activity (mU/mg protein) was determined by measuring the decrease in absorbance at 340 nm caused by nicotinamide adenine dinucleotide phosphate (NADPH) oxidation [30]. This compound is necessary to regenerate the oxidized glutathione produced by GPx enzyme when H_2_O_2_ is reduced. The activity of SOD was determined using the Fridovich method [31] by measuring the inhibition of cytochrome c reduction induced by the xanthine/xanthine oxidase system. One unit of SOD activity was defined as the amount of enzyme that inhibits the rate of cytochrome c reduction by 50%. CAT activity was determined using the method of Beers and Sizer [32] by measuring the decrease in H_2_O_2_ concentration at 240 nm. The activity values of SOD and CAT activities were expressed as units per milligram of protein (U/mg protein).

#### 2.4.2. Oxidative Stress Markers: Malondialdehyde and H_2_O_2_ Levels

The oxidative stress status in WAT was evaluated by determining lipid oxidation levels using the method described by Draper and Hadley [33]. This method quantified the amount of malondialdehyde (MDA) (mol/g wet tissue), the end-product of the oxidative degradation of lipids, that reacts with thiobarbituric acid at 535 nm. The reactive oxygen species (ROS) levels (in the form of H_2_O_2_) were analyzed by ELISA, utilizing the “Hydrogen Peroxide Colorimetric Detection Kit” from Abnova (Taipei, Taiwan). This kit employs a color reagent to determine the amount of H_2_O_2_ spectrophotometrically at 550 nm in WAT homogenates.

### 2.5. Statistical Analysis

The results are presented as the mean ± standard error of the mean (SEM). Each group consists of six animals. Data analysis was perfomed using ANOVA (analysis of variance) in GraphPad InStat 3 (San Diego, CA, USA), to examine differences among experimental groups. Subsequently, significant differences between means were identified using the Tukey–Kramer test, considering statistically significant differences with values of *p* < 0.05.

### 2.6. Transcriptomic Analysis

#### 2.6.1. Isolation of RNA from Lipid Tissue

RNA was extracted using the RNeasy Lipid Tissue Mini Kit (Qiagen, Barcelona, Spain). Two independent samples were obtained for each condition (C, S, and NS), every one of them corresponding to tissues of three animals in equal proportions. In this way, all the animals were represented but the number of RNA samples for transcriptomic analysis was lower. Basically, RNA extraction was done by following the instructions of the manufacturer with minimal modifications. Briefly, 100 µg of WAT (approx. 33 µg of each animal) were disrupted in 1 mL QIAzol Lysis Reagent in the presence of liquid nitrogen with mortar and pestle. The treatment with DNAase was done at this moment by adding 10 µL of RNase-free DNAase (Thermofischer, Madrid, Spain) and incubating the sample for 20 min at 37 °C, followed by 10 min at 70 °C for inactivation of DNAase. After that, the extract was applied to the spin column and the protocol was followed as recommended in the specifications. Finally, total RNA was eluted in 30 µL RNase-free water. To do a preliminary test of RNA quality, 5 µL of each sample was electrophoresed on 1% agarose gel and the presence of good quantity and non-degraded ribosomal 18S and 28S were taken as indication of good RNA quality. RNAs were immediately frozen at −80 °C until use.

#### 2.6.2. RNA Sequencing and Quality Control

Messenger RNA was purified from total RNA using poly-T oligo-attached magnetic beads. After fragmentation, the first strand cDNA was synthesized using random hexamer primers, followed by the second strand cDNA synthesis using dTTP for non-directional library. The sequencing library was ready after end repair, A-tailing, adapter ligation, size selection, amplification, and purification using paired-end 150 bp sequencing strategy. The sequencing library quality was checked with Qubit and real-time PCR for quantification and bioanalyzer for size distribution detection. The quantified libraries were pooled and sequenced on Illumina’s NovaSeq platform (Novogene, Cambridge, UK).

Low quality bases and adapter contamination were removed with the fastp toolkit [34,35]. A summary of QC data is show on Table S1 of Supplementary Material.

#### 2.6.3. Reads Mapping to the Reference Genome and Quantification of Gene Expression Level

HISAT2 v2.0.5 was used for aligning reads to the *Rattus norvergicus* reference genome [36]. The mapped reads of each sample were assembled by StringTie (v1.3.3b) in a reference-based approach [37]. A summary of mapping results as well as reads distribution are shown in Table S2 and Figure S1, respectively (Supplementary Data).

FeatureCounts v1.5.0-p3 was used to count the reads numbers mapped to each gene. Afterwards, FPKM of each gene was calculated based on their length and reads count mapped to them. FPKM, expected number of Fragments Per Kilobase of transcript sequence per Millions base pairs sequenced, considers the effect of sequencing depth and gene length for the reads count at the same time, and is currently the most commonly used method for estimating gene expression levels [38].

#### 2.6.4. Differential Gene Expression Analysis

After gene expression level quantification, statistical analysis of the expression data was done using DESeq2 (version 1.20.0) to screen genes whose expression levels are significantly different [39,40]. DESeq2 provide statistical routines for determining differential expression in digital gene expression data using a model based on the negative binomial distribution. The resulting *p*-values were adjusted using the Benjamini and Hochberg’s approach for controlling the false discovery rate. Genes with an adjusted *p*-value ≤ 0.05 found by DESeq2 were assigned as differentially expressed. All analyses were done on 2 biological samples and 12 million quality controlled paired end reads. Expression levels within biological replicates for the same tissue were highly correlated (mean: 93.8%, range 92.1–94.6%). File “gene_count.xls” shows the summary of reads counts and Supplementary Figures S2–S4 show data distribution, inter-sample correlation data, and principal component analysis (Supplementary Data).

#### 2.6.5. Functional Analysis

ClusterProfiler [41] software (version 4.6.0) was used for enrichment analysis, including Gene Ontology enrichment (GO), DO Enrichment, KEGG, and Reactome database Enrichment. GO enrichment analysis of differentially expressed genes with corrected *p*-value less than 0.05 were considered significantly enriched. The clusterProfiler R package (version 4.6.0) was used to test the statistical enrichment of differential expression genes in KEGG pathways (http://www.genome.jp/kegg/ (1 November 2023)). For KEGG (Kyoto Encyclopedia of Genes and Genomes) enrichment analysis, pathways with padj value less than 0.05 were considered significant.

## 3. Results and Discussion

**Figure 1:**
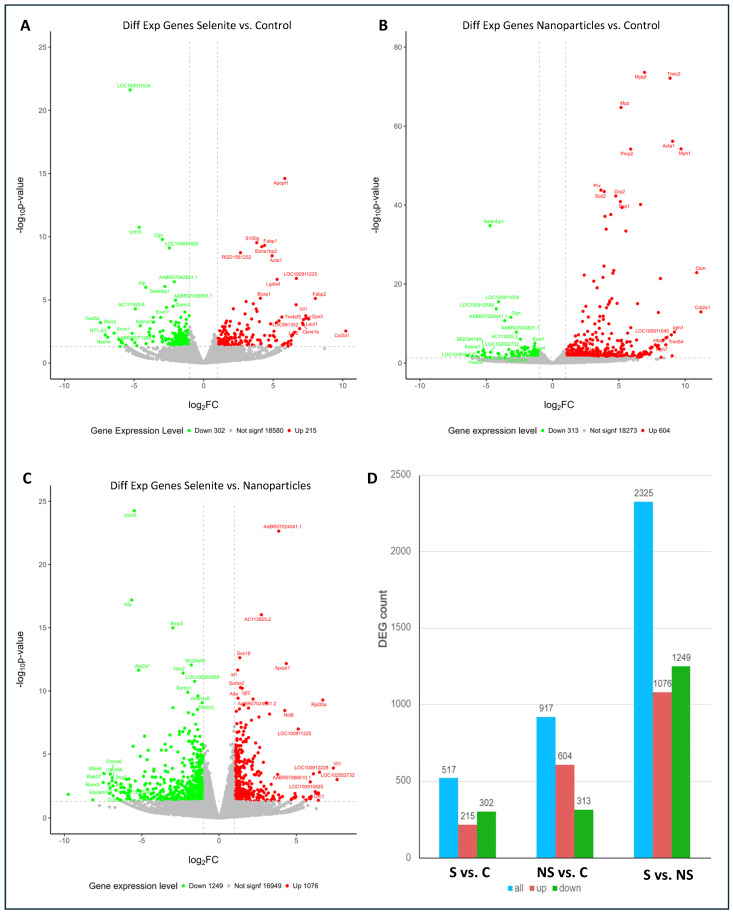
“Volcano plots” showing the distribution of the differential gene expression level (Log_2_FC (fold change)) vs. their significance (Log10 *p*-value) in all the different pairwise combinations: (**A**) S vs. C; (**B**) NS vs. C; and (**C**) S vs. NS. (**D**) Number of genes differentially expressed between S vs. C; NS vs. C; and S vs. NS. Experimental groups: C, control group; S, selenite group; NS, SeNPs group.

Figure 1 summarizes the overall analysis of gene expression changes between different conditions. Figure 1D shows the number of genes whose expression changed in all pairwise comparisons. After comparing the changes in gene expression levels between S and C, it could be seen that 18,580 genes did not significantly alter it, whereas 517 genes (2.7%) did, of which 302 (1.58%) were downregulated, and 215 (1.12%) were upregulated (Figure 1A,D). Treatment with SeNPs provoked a stronger alteration in the expression pattern when compared with the control group: while 18,273 genes did not show significant altered expression, 917 genes (4.75%) were affected by the nanoparticle’s exposure, of which 313 (1.62%) were downregulated and 604 (3.13%) showed increased expression (Figure 1B,D).

The most outstanding changes were observed after comparing both forms of Se treatment. This revealed a total number of 16,949 genes without alterations, while 1249 (6.48%) dropped their expression levels and 1076 (5.58%) showed upregulation, totaling 2325 (12%) genes whose expression was modified (Figure 1C,D).

Volcano plots show the overall distribution of fold changes of differentially expressed genes and their significance (Figure 1A–C). Treatment with selenite vs. control group led to changes in gene expression levels ranging from −6 to 10 log_2_FC (fold change) (Figure 1A). Relative to signification, the higher −log_10_ *p* value reached was 22; however, most of these values were between 1.5 and 10. On its side, the treatment with SeNPs produced expression changes ranging from −6 to 11 regarding the control (Figure 1B). In this case, the higher −log_10_ *p* value was 74, being six genes around 60 values, all of them upregulated. However, most of these genes reached a *p* value between 1.5 and 40, indicating that SeNPs treatment leads to higher significant gene changes than selenite treatment. The comparison of S vs. NS showed genes with 10-fold reduction in their expression levels to 8-fold overexpression (Figure 1C). The maximal *p* value reached in this comparison was 24, with most of these genes having a *p* value around 1.5 to 15.

**Figure 2:**
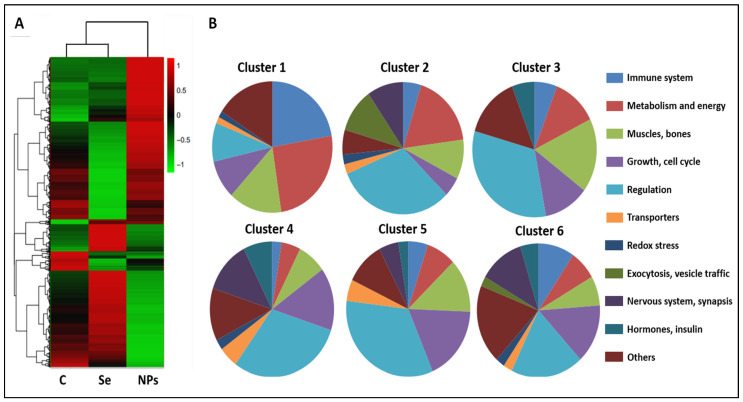
Gene clustering according to their expression patterns. (**A**) Control and Se groups showed much more similar profiles, while NPs displayed a completely different pattern of gene expression. (**B**) Genes were grouped in six clusters in which twelve main categories were identified according to cellular processes or organs. Experimental groups: C, control group; S, selenite group; NS, SeNPs group.

Genes clustering was performed in order to see whether they grouped according to their expression patterns (Figure 2A). The first conclusion was the overall comparison between the three groups; the control group exhibited greater similarity to the S group, whereas the SeNPs-treated group was much more dissimilar. Moreover, the algorithm grouped the genes in six clusters according to their patterns of expression.

Cluster 1 grouped genes that showed a low level of expression in C and S and a high level of expression in NS (Figure 2B). This cluster is quite interesting since it reveals differences in the genes’ expression related to the form of selenium application. It was characterized by the high representation of two main categories, namely the immune system and the metabolism/energy/mitochondrial function, which were highly overexpressed in the NS group. Among these genes, we could find some involved in catabolism and protein degradation as well as mitochondrial function. In addition, some genes related to the immune system were detected in this cluster (prostaglandin receptor, interleukin 1 receptor, and chemokine). These results could suggest a mobilization of resources for activation of the immune response.

Cluster 2 grouped genes were repressed in the S group regarding the other two treatments. The most outstanding characteristics were the diminution of genes involved in the immune response and the increase in genes involved in regulation. In addition, two categories emerge, including vesicles traffic and genes related to the nervous system, in particular, to motor neurons.

Cluster 3 was the one containing the lowest number of genes, 38, and they were downregulated in the control group. Besides a high percentage of regulatory genes, the category most represented was that including genes involved in cytoskeleton, muscles, and bones growth and development.

Cluster 4 contained genes highly overexpressed with the selenite treatment, whereas the expression in the C and NS groups is low. Again, this cluster is important since it reflects differences due to the form of administration. The high proportion of genes related to the growth and cell cycle, together with those involved in nervous system and synapsis, was noteworthy. In addition, some genes involved in hormone signaling were identified.

Cluster 5 included genes with high expression in the C group and repressed in both selenium treatments, particularly in S rats. In this particular group, this cluster is similar to cluster 3, since the differences seem to be due to the administration of selenium, regardless of the form. Many of the genes included in this cluster were related to cytoskeleton, muscles, growth, and cell division.

On its side, Cluster 6 grouped genes that were highly repressed with SeNPs treatment, whereas in the C and S groups, the expression was high. This cluster again reflects differences between the S and NS groups, which are related to the administration form. The gene *Eva-1* predicted to be involved in apoptotic process and autophagy stood out among the ones included in this cluster, since it could be correlated to lesser adipose tissue in SeNPs treated rats.

It is worth noting that a small but significant representation of genes related to redox balance were identified in clusters 1, 2, 4, and 6. This aspect will be studied in detail in the next sections.

**Figure 3:**
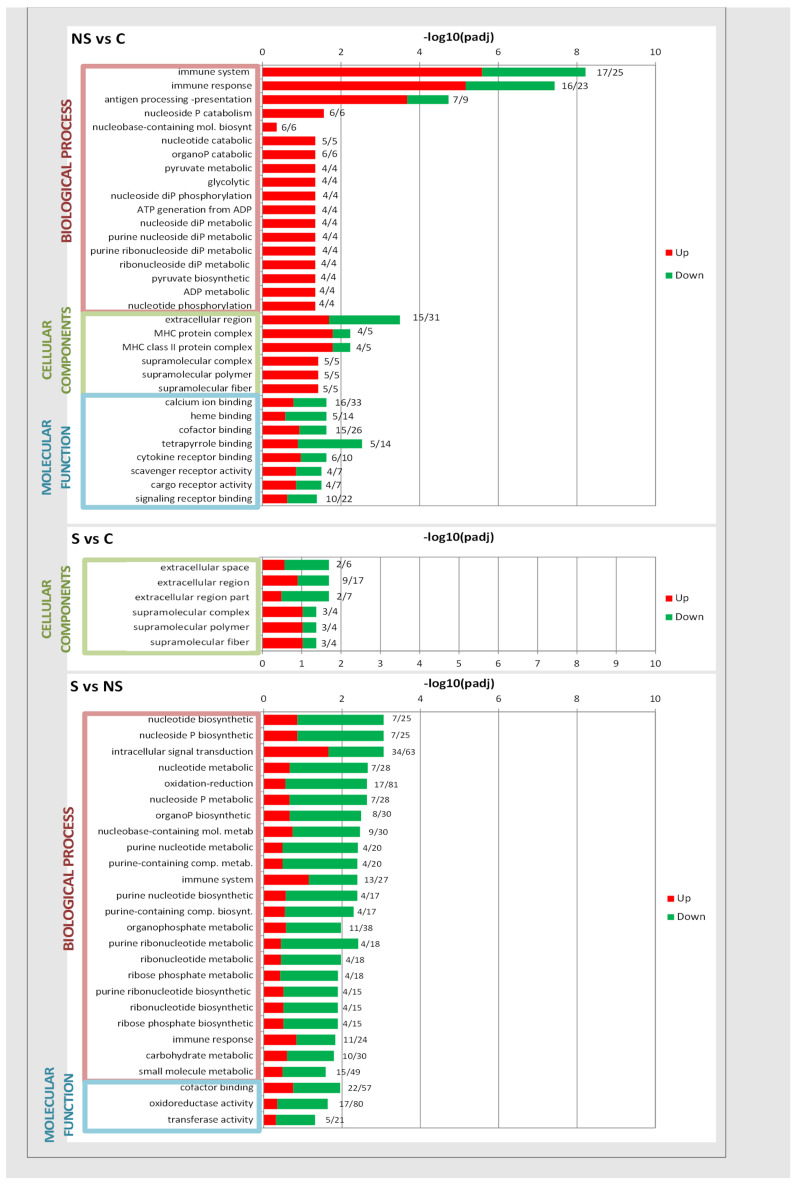
Gene Ontology (GO) enrichment analysis with upregulated (red) and downregulated (green) genes, divided by significant terms and according to the three major categories: biological processes, cell components, and molecular function. Inordinate axis was represented in GO Term’s level of significance of enrichment, expressed as −log_10_(padj). At the end of each bar, the number of upregulated genes/the total number of affected genes appeared. Experimental groups: C, control group; S, selenite group; NS, SeNPs group. The figure shows the different pairwise combinations: NS vs. C; S vs. C; and S vs. NS.

Figure 3 represents the GO terms with significant enrichment between every two conditions. Treatment with SeNPs in adolescent rats caused a higher number of significant enrichment GO terms when compared with those of group C or S. Among the top 32 GO terms found in the comparison of NS vs. C, most of them were upregulated, being especially significant (padj = 8), the GO branch genes of biological process (BP) related to the immune system and response (17 or 16 upregulated genes vs. 25 or 23 total genes, respectively). This treatment significantly affected 18 terms from BP, 6 terms from cellular components (CC), and 8 terms from molecular function (MF). Selenite treatment only significantly affected six terms from CC. Therefore, when comparing selenite vs. SeNPs, there were no differences in the CC category. However, selenite affected 23 terms from BP and 3 terms from MF, with most of them being downregulated.

In this last comparison, among the 26 highlighted GO terms analyzed, it was observed that selenite treatment provoked an extremely negative regulation in genes involved in oxidation–reduction and oxidoreductase activity (64 or 63 downregulated genes vs. 81 or 80 total genes, respectively), and intracellular signal transduction (34/63) and cofactor binding (22/57) showed that selenite compared with SeNPs affects a great number of genes implicated in these functions. Probably, the differences in the effect on WAT of these treatments reside in these genes.

**Figure 4:**
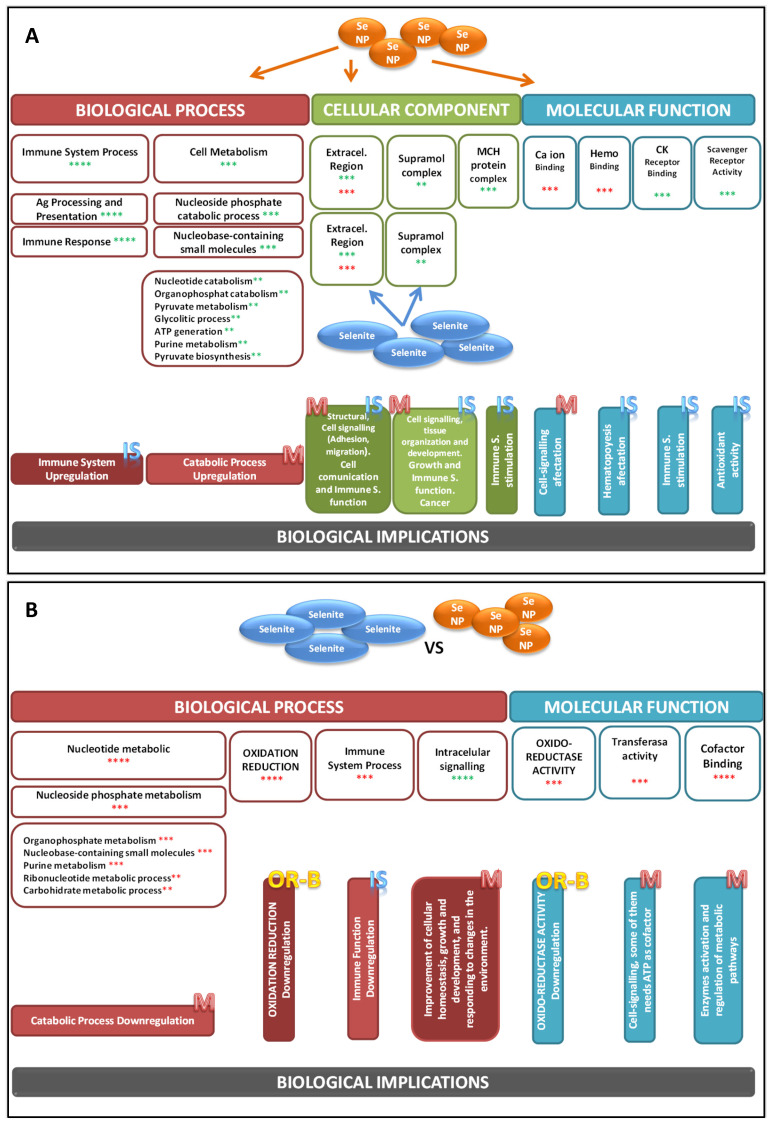
Biological functions of selenite and SeNP in WAT, including the visualization of Gene Ontology (GO) terms for differentially expressed genes, along with their signification and regulation. (**A**) Biological function of NS vs. C, and S vs. C on WAT. (**B**) Biological function of S vs. NS on WAT. The figures include the three main GO branches: biological process, cellular component, and molecular function. Experimental groups: C, control group; S, selenite group; NS, SeNPs group. Signification: ** *p* < 0.01, *** *p* < 0.001, **** *p* < 0.0001. Green stars: upregulation, red stars: downregulation. IS: immune system function, M: metabolic function, OR-B: oxidative–reduction balance.

For all this, Figure 4 shows the main biological functions classified by GO terms of selenite and SeNPs treatments in WAT. Figure 4A shows that compared to control adolescent rats, SeNPs-exposed animals mainly presented an upregulation in the genes related to BP, especially powerfully those related to immune system (IS) response (*p* = 1.8 × 10^−11^), antigen processing and presentation (*p* = 1.7 × 10^−7^), and immune response (*p* = 2.2 × 10^−10^); this treatment also increased those genes related to cell metabolism, mainly those related to catabolic pathways. SeNPs supplementation also activated cellular components’ GO branch genes, mainly modifying the extracellular region (*p* = 7.7 × 10^−6^), and increased supramolecular complex and MCH protein complex functions, which were involved in metabolic and IS functions. Finally, SeNPs supplementation affected some genes included in the MF GO branch, downregulating those related to Ca ion binding and hemo binding functions, and upregulating Ck-receptor binding and scavenger–receptor activity, indicating once more the deep relationship among SeNPs treatment and IS activity. Selenite supplementation only modified those genes implicated in the CC GO branch. Similarly to SeNPs treatment, it modulated the extracellular region and upregulated the supramolecular complex area, increasing selenite treatment cell communication and growth. Figure 4B compares BF of selenite vs. SeNPs treatment, showing that SeNPs upregulated the genes implicated in BF, especially powerfully those related to nucleotide metabolism (*p* = 2.0 × 10^−5^) and oxidative reduction balance (OR-B) (*p* = 2.6 × 10^−5^), but also the IS process; however, intracellular signaling genes were deeply downregulated (*p* = 5.8 × 10^−6^). These results imply that selenite has lower effects than SeNPs on catabolism, OR-B, and IS; nevertheless, it improved the growth and development process. Relative to MF, SeNPs increased oxido–reduction activity, transferase activity, and dramatically affected the cofactor binding function (*p* = 3.7 × 10^−5^). So far, selenite had lower effects on OS balance, ATP generation, and the catabolic process.

Table 1 shows that the three experimental groups of adolescent rats presented similar weight at the end of the experimental period. However, only NS rats had lower relative WAT mass (*p* < 0.05) than C ones, despite the fact that both supplemented groups received the same amount of extra-Se (*p* < 0.01). In spite of GPx activity being similarly increased after both supplemented treatments (*p* < 0.01), the activities of SOD and CAT and the SOD/CAT-GPx ratio were significantly enhanced in NS rats vs. C and S ones (*p* < 0.001). Finally, in WAT, the amount of H_2_O_2_ (*p* < 0.01) and MDA (*p* < 0.05) levels were increased in NS animals vs. C ones. The amount of H_2_O_2_ was also higher in NS vs. S rats (*p* < 0.05). Taking all these data together, it is demonstrated that both Se treatments increased the amount of Se ingested and the activity of the selenoprotein GPx in WAT in the same way. However, depending on the treatment used, the rest of antioxidant enzymes activities are differently affected. Selenite supplementation did not modify SOD or CAT activities, and did not alter the amount of the ROS H_2_O_2_ or lipid oxidation. By contrast, SeNPs administration significantly increased the antioxidant activity of SOD and CAT; even more, H_2_O_2_ levels and MDA values were increased indicating that oxido–reduction activity is highly activated, together with lower WAT mass. For this reason, it will be very interesting to analyze the genes related to oxido–reduction response which could be changed depending on Se treatment.

In this context, Table 2 shows the list of upregulated and downregulated genes as a result of selenite supplementation compared to SeNPs, highlighting significant differences in expression within the oxidation–reduction process from the BP GO branch in the S vs. NS groups. In this process, 81 genes were expressed in a significantly different way. The same genes (80) were also observed in an oxidoreductase activity process from the MF GO branch, being the processes with the largest number of modified genes by far when S and NS groups are compared. This demonstrates that supplementation with selenite or SeNPs plays an essential role that is deeply established in the oxidation–reduction system [42], as these forms of Se administration have the capacity to influence not only the activity of antioxidant enzymes and lipid oxidation, as shown in Table 1, but also the genetic expression of 80 genes involved in oxidative balance in the WAT of adolescent rats. This agrees with previous studies using this same experimental model, which showed that both forms of Se supplementation affected GPx1 even at the transcriptional level, since they enhanced the expression of this Se-dependent enzyme in WAT in the same way [7]. However, regarding the genes involved in these processes, treatments with selenite and SeNPs acted differently.

Specifically, selenite treatment vs. SeNPs upregulated 17 genes mainly related to oxidative stress and energy metabolism, with *Cyp26b1* exhibiting the highest change (Log_2_FC = 1.57). This gene encodes Cytochrome P450 Family 26 Subfamily B Member 1, which is involved in drug and retinol metabolism, synthesis of cholesterol and other steroids, and presents oxidoreductase activity, acting as a paired donor that reduces oxygen. Curiously, rats supplemented with selenite also showed a positive regulation of one selenoprotein, the GPx3, compared to SeNPs-treated rats (Log_2_FC = 0.80). Recent studies have found that selenite treatment increases GPx3 mRNA and protein levels in a dose-dependent manner, enhancing insulin receptor (IRS-1) expression through the activation of the transcription factor Sp1 in different adipocyte cell culture models [43,44]. This improvement in insulin sensitivity increases the differentiation and function of adipocytes. Similar results were previously found in adolescent rats supplemented with oral bulk sodium selenite [7], which showed increased pancreatic and normal WAT mass, higher insulin serum levels, normal glucose serum values, and increased IRS-1 WAT expression. This supports the notion that this supplementation promotes adipogenesis and insulin sensitivity, leading to general anabolism without obesity or inflammation. Interestingly, selenite also highly upregulated the expression of the gen *Ptgs1*. The *Ptgs1* gene encoded the protein prostaglandin-endoperoxide synthase 1 also known as Cyclooxygenase 1 (COX-1). This is a key enzyme in prostaglandin biosynthesis since it converts arachidonic acid to prostaglandin (PG) H2. This reaction involves both cyclooxygenase (dioxygenase) and hydroperoxidase (peroxidase) activity. There are two isozymes of COX encoded by distinct gene products: a constitutive COX-1 (encoded by *Ptgs1* gen) and an inducible COX-2 (encoded by *Ptgs2* gen), which differ in their regulation of expression and tissue distribution. The expression of these two transcripts is differentially regulated by relevant cytokines (COX-2) and growth factors (COX-1) [45]. In general terms, functionally, COX-1 is involved in cell signaling and maintaining tissue homeostasis. Specifically in WAT, COX-1 is considered as an important modulator of adipogenesis [46]. This upregulation of *Ptgs1* gen after selenite treatment could be one of the mechanisms by which selenite improves adipogenesis.

On the other hand, compared to SeNPs treatment, selenite caused the repression of 64 genes involved in energy metabolism (35), oxidative stress (14), mitochondrial respiration (7), and others’ process (8). Consequently, these same genes were significantly upregulated in adolescent rats treated with SeNPs, which was also in line with the results previously obtained regarding the oxidative balance in the WAT of these rats. Most of these genes (around 42 (35 + 7)) encode proteins and enzymes that participate in catabolic pathways such as glycolysis, gluconeogenesis, the pentose-phosphate pathway, the pyruvate and TCA cycle, and amino acid metabolism, as well as in the mitochondrial electron transport chain. Therefore, most of them were involved in degradative metabolism (catabolism of lipids, sugars, and proteins) and mitochondrial respiration, followed by genes involved in stress management. All these processes lead to the production of energy for the cell, activating mitochondria function, generating a high amount of ROS, and contributing to the high oxidative–reduction response observed in the WAT of NS rats. Furthermore, SeNPs supplementation increased the expression of an additional 14 genes related to the regulation of oxidative stress. In this sense, the *Msra* gene, which encodes methionine sulfoxide reductase, showed the greatest enrichment compared to the S group (Log_2_FC = −1.53). This protein catalyzes the reversible oxidation–reduction of methionine sulfoxide and plays an important role in repairing proteins that have been inactivated by oxidation. Additionally, we also observed a significant increase in the expression of *Sod2* although its fold change was not as high (Log_2_FC = −0.43). However, the upregulation for the activity of this enzyme, which destroys free superoxide radicals that are toxic to biological systems, had a major impact in the WAT of NS adolescent rats since it exhibited a markedly higher antioxidant activity compared to the other groups (Table 1), indicating an important antioxidant posttranscriptional regulation. This effect also occurs when CAT activity was analyzed. Finally, SeNPs also upregulated the gen *Ptgs2*. The *Ptgs2* gene encoded the inducible COX-2, which converts arachidonic acid into different PGs, including PGD2, PGE2, PGF2α, and PGI2. This enzyme is usually induced by inflammation and leads to an immune response. However, ROS are also important inductors of this enzyme; moreover, COX-2 also contributes to generate ROS [47]. Specifically in adipocytes, COX-2 was the major isoform involved in mediating negative effects on adipogenesis [48]. In fact, COX-2 and PGE2 are related to lipolysis induction in adipocytes [49]. Contrary to what might be supposed, these effects are related to autophagy, anti-inflammation process, and metabolic benefits, since it is demonstrated that adipocyte COX-2 and PGE2 are key regulators of FoxP3+ regulatory T cells (Tregs) proliferation during intermittent fasting (a mode of induced adaptative autophagy) leading to an anti-inflammatory response and metabolic benefits [50]. Since in adipocytes it is well known that ROS stimulates the inducible form COX-2 [51], which in turn stimulates Tregs proliferation, which have an important role in controlling adipocyte inflammation [52], and is also involved in lipolysis, this increase in the *Ptgs2* gene after SeNPs administration could be an important link between SeNPs–ROS–immune system stimulation and lipolysis in WAT.

**Figure 5:**
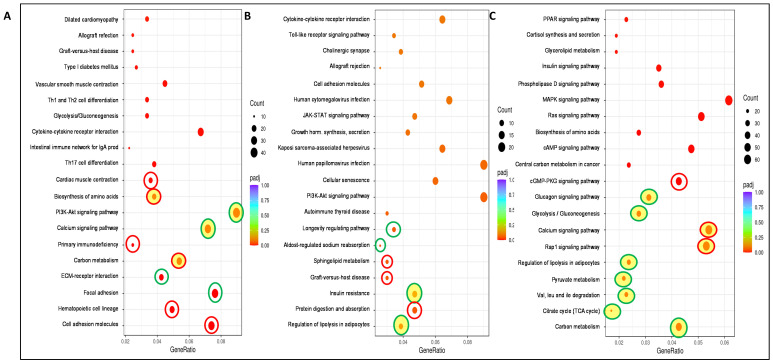
Main metabolic pathways affected in WAT after the treatments by using KEGG pathway enrichment dot-plot diagrams. (**A**) represents NS vs. C rats, (**B**) represents S vs. C rats, and (**C**) represents S vs. NS rats. The dot-plot diagram represents the top 20 signaling pathways regulated by the treatments. X-axis represents the ratio of enriched target genes/background genes. Y-axis represents the term of enriched pathways. The sizes of the dots indicate the number of target genes in a certain pathway, and the colors of the dots reflect the different values of padj; the lower the points are located, the more significant they are. Only the dots representing the top ten significantly enriched genes are surrounded by color rings; green rings indicate that the genes are mostly downregulated, and red rings indicate upregulation; those filled with yellow are pathways directly related to WAT metabolism and growth. Experimental groups: C, control group; S, selenite group; NS, SeNPs group.

**Figure 6:**
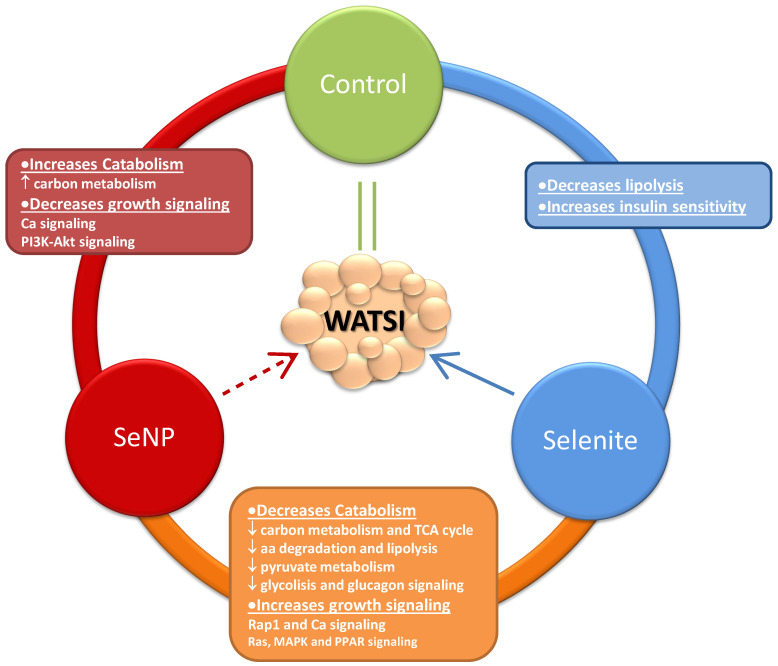
Main metabolic pathways related to WAT development and WATSI after different Se treatments, taking into account KEGG pathways enrichment results. An upward arrow indicates an increase, and a downward arrow indicates a decrease.

Relative to the implication of the different genes affected by the Se treatments used in the WAT main metabolic pathways (using KEGG pathway enrichment dot-plot diagrams), Figure 5A shows that SeNPs treatment significantly modified the 20 pathways which appeared in the image compared to C rats. Among the 10 most significant, SeNPs increased the pathways related to cell adhesion molecules, hematopoietic cell linage, carbon metabolism, primary immunodeficiency, biosynthesis of amino acids and cardiac muscle contraction, and decreased those related to focal adhesion, ECM-receptor reaction, the calcium signaling pathway and the PI3K-Akt signaling pathway, this last pathway compromising 40 target genes. When selenite was administered (Figure 5B), only 7 out of 20 pathways shown or represented in Figure 5B were significantly changed (4 downregulated and 3 upregulated), with the regulation of lipolysis in adipocytes the most affected pathway vs. C rats. Selenite decreased the regulation of lipolysis in adipocytes, the insulin resistance process, the aldosterone-regulated sodium reabsorption, and the longevity-regulating pathway, and increased protein digestion and absorption pathways, gralt-vs-host disease, and sphingolipid metabolism. Finally, when selenite treatment was compared vs. the SeNPs one, the 20 pathways shown in Figure 5C were significantly affected. Among the 10 most relevant pathways, selenite decreased 7 and enhanced 3, with 9 related to metabolism, an aspect that will be analyzed in more detail in Figure 6. Selenite mainly decreased the effect of SeNPs treatment on carbon metabolism, but also on the tricarboxylic acid (TCA) cycle, valine-leucine-isoleucine degradation, pyruvate metabolism, regulation of lipolysis in adipocytes, glycolysis/gluconeogenesis, and the glucagon signaling pathway. By contrast, selenite increased the Pap1 signaling pathway, calcium signaling pathway, and cGMP-PKG signaling pathway.

These effects of Se supplementations on WAT main metabolic pathways align with those found in oxidative balance. In this context, selenite treatment, which inhibits lipolysis and enhances growth signaling, upregulated the genes associated to the antioxidant enzyme GPx3, which improves the insulin signaling process contributing to avoid lipolysis [44]. It also increased the gene which encoded COX-1, an important modulator of growth pathways [45] and a promoter of adipogenesis [46]. Conversely, SeNPs supplementation increased the oxidative response, and oxidative genes involved in degradative and mitochondrial respiration, particularly the gene encoding COX-2. As mentioned, COX-2 in adipocytes is specifically involved in avoiding adipogenesis [48,49] and promoting lipolysis [52], in complete agreement with the WAT catabolic pathways induced by SeNPs treatments.

In this regard, Figure 6 represents a model of the results found by depicting the interrelationship between forms of selenium administration, affected metabolic pathways, and WAT. Figure 6 shows how selenite treatment improves adipocytes growth by decreasing lipolysis and increasing insulin sensitivity compared to control rats, without modifying WATSI (Table 1) [7]. By contrast, SeNPs treatment, with the same dose of Se administration as selenite, led to a lower relative WAT mass (Table 1) by increasing catabolism via carbon metabolism, and decreasing growth signaling pathways such as calcium-signaling and the PI3K-Akt pathway. However, the most relevant data to understand the difference among these treatments on WAT development are observed when comparing selenite to SeNPs treatments. In this case, it is quite clear that both treatments have opposite effects in WAT development; selenite collaborates with adipocytes growth and SeNPs to WAT catabolism affecting several pathways. In this context, selenite increased growth signaling pathways such as Rap 1 and calcium signaling and Ras, MAPK, and PPAR signaling, but also contributes to decrease the WAT catabolism induced by SeNPs; it decreased carbon metabolism, the TCA cycle, degradation and lipolysis, pyruvate metabolism, glycolysis/gluconeogenesis, and glucagon signaling pathway.

The present study has several limitations. Firstly, economic constraints led to a small sample size in the transcriptomic analysis, as WAT tissue from three animals was pooled to extract total RNA. Consequently, only two pools were used per group. However, these pools contain total RNA from all three rats, ensuring representation of all rats in the group with statistical signification. Moreover, the transcriptomics data are part of a more complete study involving other techniques such as microscopy, adipose tissue weight, redox enzymes, protein levels, etc. Indeed, the conclusions obtained from gene expression were also supported by other previous studies. However, another limitation in this study is that the identification of changes in gene expression does not always translate directly into protein activity. In this regard, additional protein expression measurement should be done.

## 4. Conclusions

Our results showed that treatment with SeNPs provoked a greater number of affected genes in WAT than in adolescent rats treated with selenite. Therefore, this treatment affects a higher number of cellular processes. Processes related to the immune system, the catabolism and mitochondrial function, and the oxidative balance. Relative to OS, SeNPs enhanced the activity of antioxidant enzymes; this increase was much higher than the level of gene expression induction, suggesting additional post-transcriptional regulation. Despite that, there was an accumulation of ROS and MDA in WAT, suggesting oxidative damage. The analysis of all genes related to OS showed that, overall, 80 genes were significantly affected in the presence of SeNPs compared to that of selenite. Most of them were involved in degradative metabolism (catabolism of lipids, sugars, and proteins) and mitochondrial respiration, followed by genes involved in stress management. Selenite treatment upregulated some genes related to oxidative stress, such as the selenoprotein GPx3 related to insulin sensitivity in WAT; this treatment also decreased lipolysis and catabolism avoiding WAT mass destruction. The analysis of KEGG pathway enrichment showed that SeNPs treatment increased main catabolic pathways and decreased an important growth signaling process, contributing to the decrease in WAT mass found in NS rats. In conclusion, the administration of SeNPs during adolescence led to enhanced catabolism, autophagia, and increased OS in WAT. Our results suggest a possible application of SeNPs in the reduction in adipose tissue and induction of the immune response. These findings provide data for dietary approaches to prevent the increased pandemia of obesity during adolescence, where WAT and inflammation are increased. Future experimental studies must be assessed before considering the application of SeNPs for its anti-obesity activity as a possible approach for this disease.

## Figures and Tables

**Table 1:** Oxidative balance in WAT at the end of experimental process. Antioxidant protein activities (SOD, CAT, and GPx), H_2_O_2_ levels, and lipid oxidation products (MDA).

	C	S	NS
**Increased body weight** (g/d)	6.01 ± 0.1	6.07 ± 0.2	5.98 ± 0.2
**Total Se intake** (µg/d)	3.48 ± 0.08	6.81 ± 0.14 **	6.59 ± 0.09 **
**WATSI** (%)	0.62 ± 0.02	0.59 ± 0.03	0.50 ± 0.02 *
**SOD activity** (U/mg protein)	12.6 ± 0.8	12.9 ± 0.3	36.9 ± 2.5 ***, ^sss^
**CAT activity** (U/mg protein)	101 ± 9	108 ± 9	184 ± 9 ***, ^sss^
**GPx activity** (mU/mg protein)	201 ± 4	254 ± 13 **	256 ± 11 **
**SOD/CAT-GPx ratio**	4.2 ± 0.1	3.5 ± 0.1	8.3 ± 0.2 ***, ^sss^
**H_2_O_2_** (µM)	73.8 ± 2.9	76.3 ± 3.9	91.9 ± 2.9 **, ^s^
**MDA** (mol/g wet tissue)	21.0 ± 1.3	26.0 ± 1.6	28.3 ± 2.1 *

The results were expressed as means ± SEMs and analyzed using a multifactorial one-way ANOVA followed by Tukey’s test. The number of animals used in each group is *n* = 6. Experimental groups: C, control group; S, selenite group; NS, SeNPs group. Significance: vs. C, * *p* < 0.05, ** *p* < 0.01, *** *p* < 0.001; vs. S, ^s^ *p* < 0.05, ^sss^ *p* < 0.001. WATSI: white-adipose-tissue somatic index; SOD: superoxide dismutase; CAT: catalase; GPx: glutathione peroxidase; MDA: malondialdehyde.

**Table 2:** List of genes related to the oxidation–reduction process significantly upregulated (in red) and downregulated (in green) when selenite vs. SeNPs treatments were compared.

Status	Related Genes with	Gene_Symbol	Log_2_FC
UPREGULATED BY SELENITE	Energy metabolism	*Me3, Ndufa5*	[0.93]–[0.55]
	Oxidative stress	*Cyp26b1* *, Aox1, GPx3, Nos3, Mt1, Msrb3,* *Cyp4b1, Cyp2e1, Cyp2ab1, Cyp27a1, Cyp2j4, LOC100912391*	[1.57]–[0.42]
	Others	*Ptgs1, Alox15, Alox12*	[1.22]–[1.07]
DOWNREGULATED BY SELENITE	Energy metabolism	*Acadsb, Hadh, Acox3, Acad10, Acadm, Acadl, Mecr, Gapdh, Ldha, G6pdx, LOC303448, Gpd1, Aldh7a1, Aldh6a1, Aldh9a1, Aldh2, D2hgdh, Ldha, Pdpr, Idh3g, Fh, Mdh2, Idh3B, Acad9, Mdh1, Pcyox1, Ivd, Dld, Hibadh, Cdo1, LOC100911156, Pycr1, Rrm2b, RGD1565368, Idh3a*	[−1.56]–[−0.37]
	Oxidative stress	*Msra, Mpo, Prdx3, Tp53i3, Aox4,* *Cyp51, Sc5d, Cyp1b1, Nsdhl, Cyp4f39, Dhcr24, Cyb5r3, Sqle, Sod2*	[−1.53]–[−0.43]
	Enzymes of the mitochondrial respiratory	*Maob, Sdha, Ndufs1, Ndufb6, LOC685596, Foxred1, Uqcrfs1*	[−1.15]–[−0.38]
	Others	*Ptgs2, Aifm1, Loxl4, Loxl2, Tas1r2,* *AC098459.1, AABR07012795.1, Aifm2*	[−2.05]–[−0.46]

The range of Log_2_FC (fold change) for the related genes is shown in the right column. The gene with the highest Log_2_FC is indicated in blue color, while the gene with the smallest Log_2_FC is highlighted in orange color.

## Data Availability

The data are not publicly available because there is still a significant amount of unpublished information.

## Supplementary Materials

The following supporting information can be downloaded at: https://www.mdpi.com/article/10.3390/antiox13060750/s1, Table S1: Data quality control summary; Table S2: Mapping of reads summary; Figure S1: The distribution of sequencing reads in the genomic region; Figure S2: Gene expression distribution for each sample; Figure S3: Inter-sample correlation heat map; Figure S4. Principal component analysis result.
