# Supplementary material for: Uncovering the Role of Selenite and Selenium Nanoparticles (SeNPs) in Adolescent Rat Adipose Tissue beyond Oxidative Balance: Transcriptomic Analysis
Source: Antioxidants (Basel). 2024 Jun 20;13(6):750. doi: 10.3390/antiox13060750 (PMC11200624; doi:10.3390/antiox13060750)

Supplementary material

Table S1. **Data quality control summary**. The table shows reads count from the raw data, with statistics of reads count for every sequencing (**raw_reads**), the base number of raw data [(number of raw reads) * (sequence length), converting unit to G] (**raw_bases**), the base number of raw data after filtering [(number of clean reads) * (sequence length)], converting unit to G (**clean_reads)**, the clean base number [(clean base=clean reads*150bp) number multiply read length, saved in G unit.] (**clean_bases**), the average sequencing error rate (calculated by Qphred=-10log10(e)) (**error_rate**), the percentage of the bases whose Q Phred values is greater than 20 [(Number of bases with Q Phred value > 20) / (Number of total bases) *100] (**Q20**), the percentage of the bases whose Q Phred values is greater than 30 [(Number of bases with Q Phred value > 30) / (Number of total bases) *100] (**Q30**) and the percentage of G&C base numbers of total bases [(G&C base number) / (Total base number)*100] (**GC_pct**).

Sample	Raw_reads	Raw_bases	Clean_reads	Clean_bases	Error_rate	Q20	Q30	GC_pct
C1	97398710	14.61G	95286486	14.29G	0.03	97.21	92.55	46.99
C2	81515354	12.23G	79873760	11.98G	0.03	97.13	92.32	49
Se1	84223368	12.63G	82452570	12.37G	0.03	97.29	92.74	46.83
Se2	90742100	13.61G	88602380	13.29G	0.03	97.07	92.26	47.37
Np1	97310696	14.6G	95444424	14.32G	0.03	97.33	92.77	48.06
Np2	86023726	12.9G	84441386	12.67G	0.03	97.68	93.66	48.42

Table S2. **Mapping of reads summary**. The table shows the comparison of reads alignments. **Total_reads**: Total clean reads used for analysis. **Total_map**: Number and percentage of reads aligned to the genome, the ratio should higher than 70%, total mapping rate: (mapped reads)/(total reads)*100. **Unique_map**: Number and percentage of reads aligned to the unique position of the reference genome (for subsequent quantitative data analysis), unique mapping rate: (uniquely mapped reads)/(total reads)*100. **Multi_map**: number and percentage of reads aligned to multiple locations in the reference genome, multiple mapping rate: (multiple mapped reads)/(total reads)*100. **Read1_map**: Number and percentage of read1 aligned to the reference genome. **read2_map**: Number and percentage of read2 aligned to the reference genome. **positive_map**: Number and percentage of reads aligned to the positive chain of the reference genome. **Negative_map**: Number and percentage of reads aligned to the negative chain of the reference genome. **Splice_map**: Number of spliced reads on the genome and its percentage. **Unsplice_map**: Number of complete reads aligned to genome and its percentage. **Proper_map**: Number of paired read1 and read2 aligned to the genome and its percentage.

sample	total_ reads	total_ map	unique_ map	multi_ map	read1_ map	read2_ map	positive_ map	negative_ map	splice_ map	unsplice_ map	proper_ map
C1	95286486	90451863 (94.93%)	83278610 (87.4%)	7173253 (7.53%)	41451864 (43.5%)	41826746 (43.9%)	41558885 (43.61%)	41719725 (43.78%)	22581798 (23.7%)	60696812 (63.7%)	80036214 (84.0%)
C2	79873760	76063029 (95.23%)	70420003 (88.16%)	5643026 (7.06%)	35177001 (44.04%)	35243002 (44.12%)	35161054 (44.02%)	35258949 (44.14%)	25260607 (31.63%)	45159396 (56.54%)	68015664 (85.15%)
Se1	82452570	78642283 (95.38%)	72413357 (87.82%)	6228926 (7.55%)	36287361 (44.01%)	36125996 (43.81%)	36181909 (43.88%)	36231448 (43.94%)	18672864 (22.65%)	53740493 (65.18%)	69945050 (84.83%)
Se2	88602380	83932925 (94.73%)	77287723 (87.23%)	6645202 (7.5%)	38502754 (43.46%)	38784969 (43.77%)	38603359 (43.57%)	38684364 (43.66%)	20708523 (23.37%)	56579200 (63.86%)	74276002 (83.83%)
Np1	95444424	91021966 (95.37%)	84170250 (88.19%)	6851716 (7.18%)	41938639 (43.94%)	42231611 (44.25%)	42028323 (44.03%)	42141927 (44.15%)	27212196 (28.51%)	56958054 (59.68%)	81283390 (85.16%)
Np2	84441386	80980777 (95.9%)	74899503 (88.7%)	6081274 (7.2%)	37468982 (44.37%)	37430521 (44.33%)	37463904 (44.37%)	37435599 (44.33%)	24530376 (29.05%)	50369127 (59.65%)	72438344 (85.79%)

Figure S1. The distribution of sequencing reads in the genomic region. Mapped regions can be classified as exons, introns, or intergenic regions. Exon-mapped reads should be the most abundant type of reads when the reference genome is well-annotated. Intron-reads may be derived from pre-mRNA contamination or intron-retention from alternative splicing. Reads mapped to intergenic regions are mainly attributed to weak annotations of the reference genome. The distribution of sequencing reads of all samples in the genomic region is shown in the figure below. The ratios in the figure are calculated from clean bases. **Exon**: The number of bases aligned to exon regions of the genome and its proportion to the number of bases aligned to the genome. **Intron**: The number of bases aligned to intron regions of the genome and its proportion to the number of bases aligned to the genome. **Intergenic**: The number of bases aligned to intergenic regions of the genome and its proportion to the number of bases aligned to the genome.

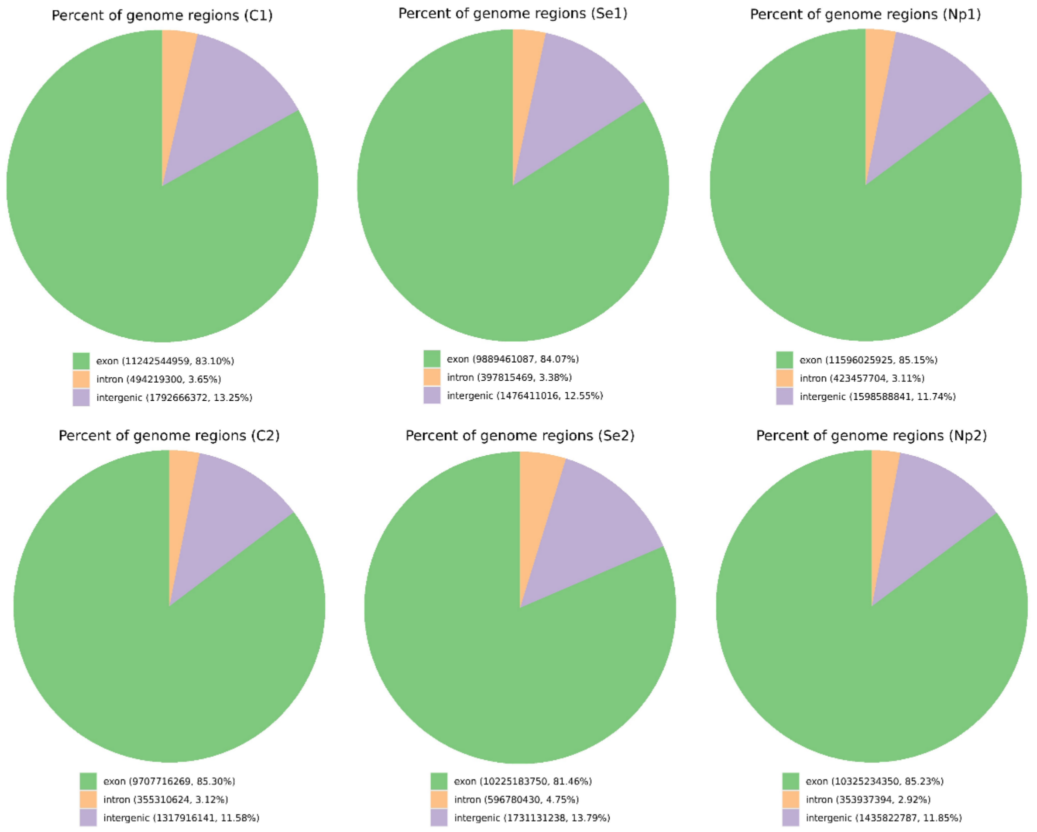

Figure S2. Gene expression distribution for each sample. To compare gene expression levels under different conditions, the distribution of gene expression levels and FPKM[8] among different samples are displayed by boxplots. X axis represents the name of the sample. Y axis indicates the log2(FPKM+1). Parameters of box plots are indicated, including maximum, upper quartile, mid-value, lower quartile and minimum.

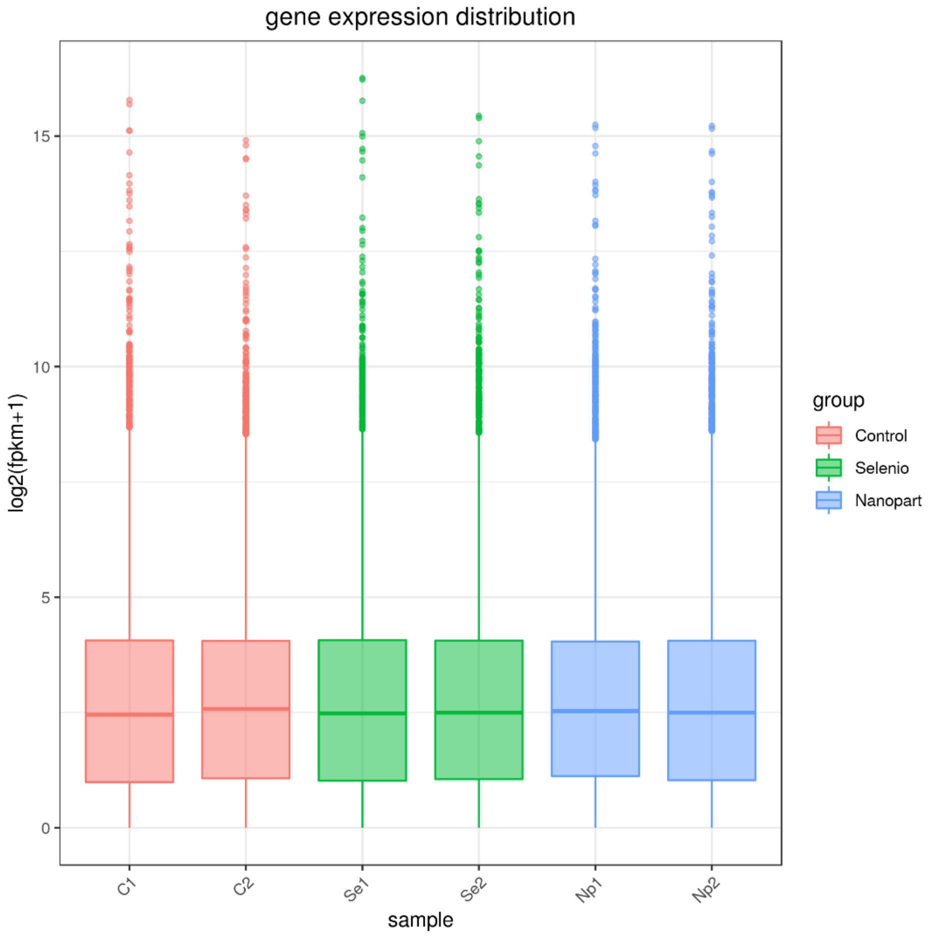

Figure S3. Inter-sample correlation heat map. Correlation of the gene expression levels between samples plays an important role in verifying reliability and sample selection. A correlation coefficient closer to 1 indicates higher similarity in expression pattern. Under ideal experiment conditions the square of the Pearson correlation coefficient should be greater than 0.92 and the R^2 should be greater than 0.8.

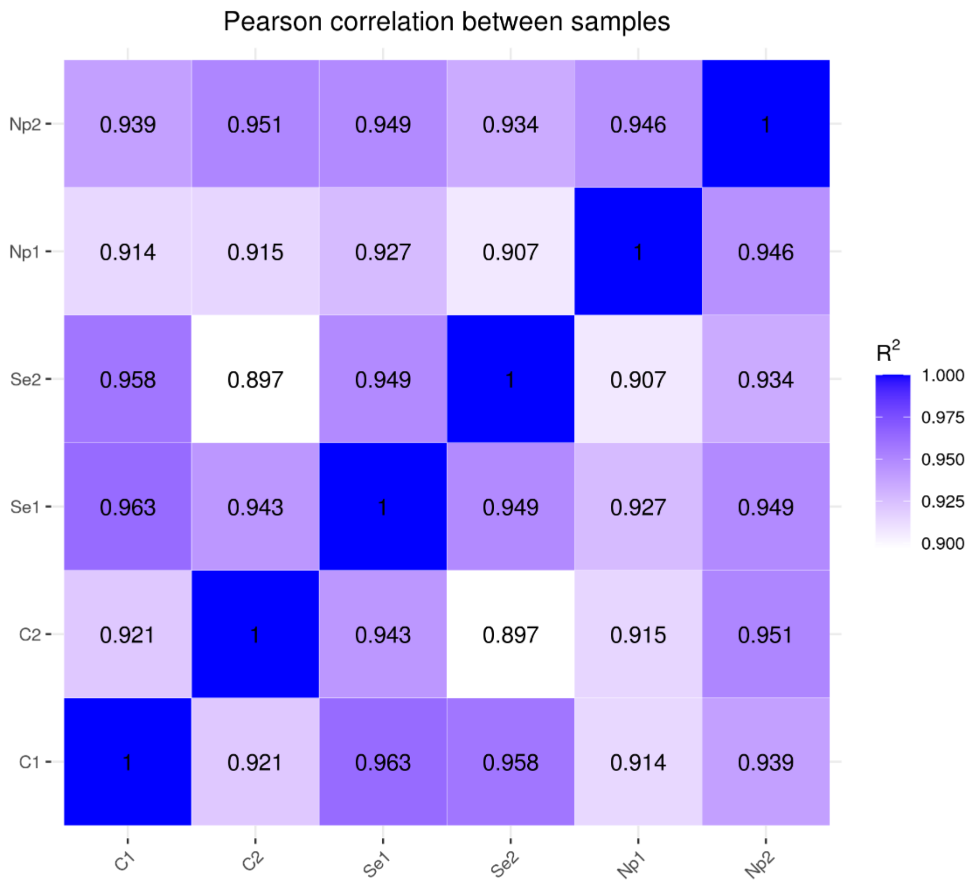

Figure S4. Principal component analysis result. The intergroup differences and intragroup sample duplication was evaluated by Principal component analysis (PCA). PCA uses the linear algebra calculation method to reduce data dimension and extract principal components. We performed PCA analysis on the gene expression value (FPKM) of all samples, as shown in the figure below. The analysis shows that control group expression pattern is closer to Selenite group than Selenium Nanoparticles group.

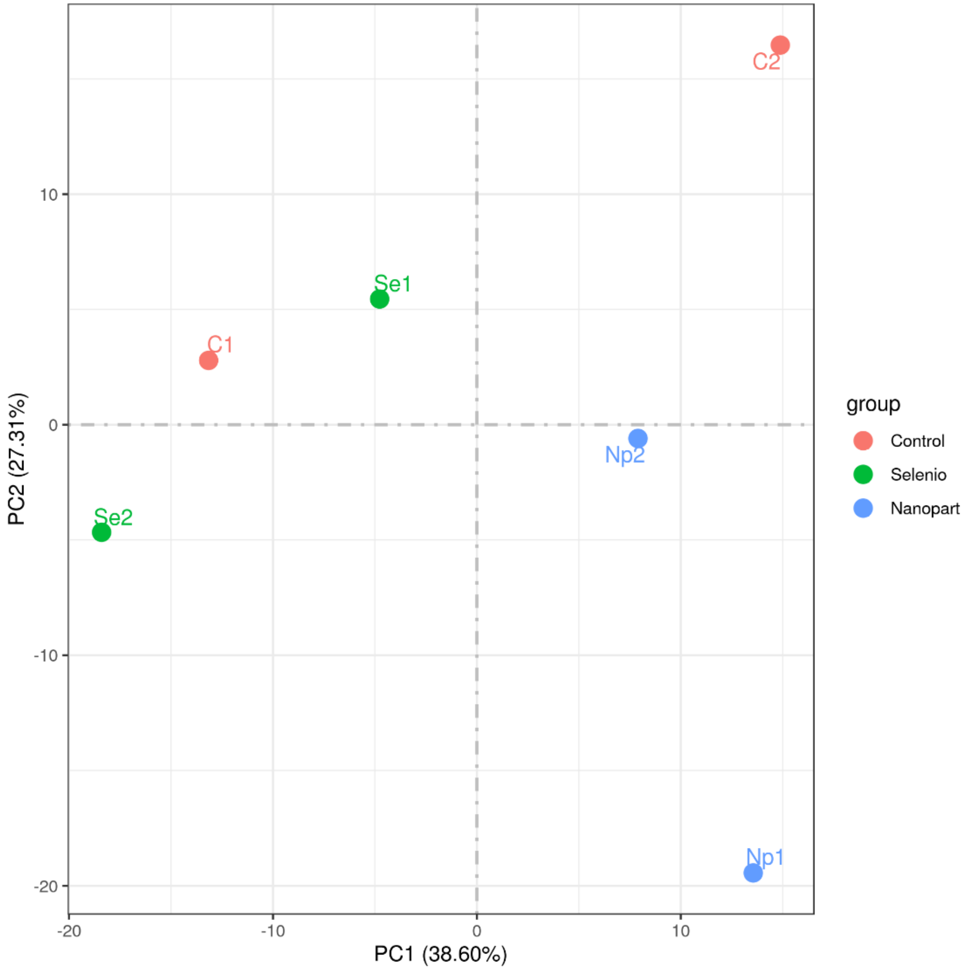

Supplement: Supplementary file 1 [file antioxidants-13-00750-s001.zip › antioxidants-3050168-supplementary.pdf]
